# Diabetes in pregnancy among indigenous women in Australia, Canada, New Zealand, and the United States: a method for systematic review of studies with different designs

**DOI:** 10.1186/1471-2393-11-104

**Published:** 2011-12-23

**Authors:** Catherine Chamberlain, Daniel Yore, Hang Li, Emily Williams, Brian Oldenburg, Jeremy Oats, Bridgette McNamara, Sandra Eades

**Affiliations:** 1 International Public Health Unit Department of Epidemiology and Preventive Medicine School of Medicine, Nursing and Health Sciences Monash University L3/89 Commercial Rd Prahan. Victoria. 3181. Australia; 2 Department of Obstetrics and Gynaecology Melbourne University PO Box 5266 Burnley. Victoria. 3121. Australia; 3 Baker IDI Heart and Diabetes Institute 75 Commercial Rd Melbourne. Victoria. 3004. Australia

## Abstract

**Background:**

Diabetes in pregnancy, which includes gestational diabetes mellitus (GDM) and type 2 diabetes mellitus (T2DM), is associated with poor outcomes for both mother and infant during pregnancy, at birth and in the longer term. Recent international guidelines recommend changes to the current GDM screening criteria. While some controversy remains, there appears to be consensus that women at high risk of T2DM, including indigenous women, should be offered screening for GDM early in pregnancy, rather than waiting until 24-28 weeks as is current practice. A range of criteria should be considered before changing screening practice in a population sub-group, including: prevalence, current practice, acceptability and whether adequate treatment pathways and follow-up systems are available. There are also specific issues related to screening in pregnancy and indigenous populations. The evidence that these criteria are met for indigenous populations is yet to be reported. A range of study designs can be considered to generate relevant evidence for these issues, including epidemiological, observational, qualitative, and intervention studies, which are not usually included within a single systematic review. The aim of this paper is to describe the methods we used to systematically review studies of different designs and present the evidence in a pragmatic format for policy discussion.

**Methods/Design:**

The inclusion criteria will be broad to ensure inclusion of the critical perspectives of indigenous women. Abstracts of the search results will be reviewed by two persons; the full texts of all potentially eligible papers will be reviewed by one person, and 10% will be checked by a second person for validation. Data extraction will be standardised, using existing tools to identify risks for bias in intervention, measurement, qualitative studies and reviews; and adapting criteria for appraising risk for bias in descriptive studies. External validity (generalisability) will also be appraised. The main findings will be synthesised according to the criteria for population-based screening and summarised in an adapted "GRADE" tool.

**Discussion:**

This will be the first systematic review of all the published literature on diabetes in pregnancy among indigenous women. The method provides a pragmatic approach for synthesizing relevant evidence from a range of study designs to inform the current policy discussion.

## Background

Gestational diabetes mellitus (GDM) is defined as "any degree of glucose intolerance with onset or first recognition during pregnancy" [1]. GDM can be a temporary glucose intolerance, as a result of hormonal changes in pregnancy, or type 2 diabetes mellitus (T2DM) that has not previously been diagnosed. Diabetes in pregnancy (DIP) includes both GDM and T2DM, which may or may not have been previously diagnosed, and type 1 diabetes mellitus.

DIP is associated with increased risks at birth for the mother (caesarean section)[2] and the infant (macrosomia, hypoglycaemia) [2]. Women with GDM are at high risk for T2DM after pregnancy [3,4], and GDM is often identified as an early step in the "natural history" of the progression to T2DM. Children born to women with DIP have higher risks for obesity and for T2DM in later life [5,6]. These observations have led to the proposal that DIP is a major contributor to the high observed prevalence of T2DM in indigenous populations and to the increasing prevalence of obesity and diabetes among their children [7]. Indigenous women in Australia, Canada, New Zealand and the United States have experienced rapid changes from a traditional diet and lifestyle, to one rich in processed, carbohydrate-dense foods and reduced energy expenditure, which are associated with increased rates of obesity, GDM and T2DM [8].

T2DM is a serious metabolic disorder, in which blood sugar is no longer controlled, and can lead to heart disease, stroke, renal disease, kidney failure, amputations and blindness [9]. The prevalence of T2DM is increasing in line with increasing rates of obesity across the globe. Indigenous populations generally have significantly higher rates than non-indigenous people within the same country, suggesting that indigenous populations have progressed past the pre-diabetes stage in this metabolic disorder [10].

The current recommendations for screening and diagnosis of GDM were written over 40 years ago by adapting methods for screening non-pregnant women in order to identify those at high risk for T2DM after pregnancy [2]. Recently demonstrated clear associations between any degree of hyperglycaemia and adverse pregnancy outcomes [11] has, however, led to a review of these long-established criteria [12]. There is ongoing controversy about use of universal or selective screening, the timing and the type of tests [13-15] and the choice of interventions for prevention management and follow-up, particularly for indigenous communities [16]. However, there appears to be consensus that women in populations with a high prevalence of T2DM should be offered screening for GDM early in pregnancy, before an oral glucose tolerance test at 24-28 weeks gestation (as is current practice), particularly if metabolic testing in this age group is not commonly conducted [17,18].

The aim of population-based screening is to reduce the burden of disease in the community by earlier detection of disease, thus providing an opportunity for intervention and improvement of health-related outcomes [19]. Early detection of GDM provides an opportunity to offer support and treatment to reduce or manage hyperglycaemia and the associated risks in both pregnancy and the long term. Pregnancy is an opportune time to offer support to women, as they have frequent scheduled contacts with health-care providers and are often highly motivated to adapt their behaviour to improve the health of their infant. In addition, any effective interventions could affect the health of the whole family, including the unborn child [20,21].

A number of factors should be considered, however, before introducing population-based interventions, including; the prevalence and natural history of the condition, current screening practice and rates, acceptability (preferences and values), efficacy and cost and the availability of adequate treatment pathways and follow-up [22]. In addition to these routine criteria, there are specific considerations related to screening in pregnancy and in indigenous populations. Increased diagnosis in pregnancy of a medical condition in a generally healthy population can be associated with unnecessary stress [23], and ineffective therapy might be given that initiates a "cascade of interventions" [24] which interfere with the normal process of pregnancy and birth. Indigenous communities in particular face a range of exacerbated health challenges, with complex cultural and social issues, which should be considered in any medical intervention [25]. The current consensus makes it likely that indigenous women will experience changes in screening practice earlier than low-risk population groups. However, the evidence that the criteria for population-based screening are met for this population group has not been reviewed, nor have the specific issues unique for this population group.

The evidence for these factors will be generated by studies with a number of different designs, including epidemiological studies, qualitative studies, measurement studies, intervention studies and even opinion pieces. Systematic review offers a rigorous process for appraising the quality of evidence; however, such reviews are often restricted, as they include only a limited number of study designs in order to focus on the internal validity of the design and not to consider the external validity or generalizability of the results for the intended purpose [26]. Thus, researchers and journal editors face challenges in making research relevant for decision-making [26]. These challenges are familiar to clinicians developing evidence-based practice guidelines, and a number of tools have been developed to "GRADE" and present evidence that may be useful for adaptation in the public health setting [27].

Our aim in this paper is to describe a method for systematically reviewing all published studies relevant to informing future discussions about screening for DIP among indigenous women in Australia, Canada, New Zealand and the United States.

## Methods/Design

### Inclusion criteria

The inclusion criterion will be broadly defined as "any publication that includes analysis or discussion of diabetes in pregnancy among indigenous women in Australia, New Zealand, Canada or the United States".

This broad definition was used so that all studies would be included and information to address the relevant screening criteria [22] could be extracted. This includes information regarding perspectives and acceptability, which affect the overall sensitivity of screening interventions when applied at a population level. The body of literature therefore comprises all original intervention, measurement and descriptive studies, as well as reviews, program descriptions and opinion pieces. Indigenous women in Australia, Canada, New Zealand and the United States were grouped, as they are considered to have similar (but not identical) social and health experiences of colonisation and rapid transition from a traditional diet and lifestyle to one of relative poverty in a high-income country [25,28,29]. In most published studies, indigenous women have been compared with non-indigenous women in the same countries, or evidence for non-indigenous women was applied to indigenous women, with arguable external validity.

### Search method

We will search the Cochrane Database of Systematic Reviews (1995-July 2010), Medline (1950-July 2010), Embase (1949-July 2010), CINAHL (1937-July 2010), and PsychINFO (1905-July 2010). The following key words in the titles and abstracts and subsequent MeSH headings will be searched:

pregnan* or antenatal or prenatal, or neonatal or newborn or infan* or fetal or fetus or foetal or foetus

AND diabet* or hyperglyc?mi* or glucose intoleran* or obes* (or gestational diabet*)

AND aborigin* or indigen* or native* or first nation*

See table 1 for a full search tree in Medline. We will not apply any language or other restrictions.

**Table 1:** Medline MeSH and key word search strategy (Same strategies used for Embase, Cinahl, Psychinfo)

1. pregnan*.mp. [mp = title, original title, abstract, name of substance word, subject heading word, unique identifier]
2. exp Pregnancy/or exp Pregnancy Outcome/
3. antenatal.mp. [mp = title, original title, abstract, name of substance word, subject heading word, unique identifier]
4. exp Prenatal Care/or exp Prenatal Diagnosis/
5. prenatal.mp. [mp = title, original title, abstract, name of substance word, subject heading word, unique identifier]
6. exp Prenatal Nutritional Physiological Phenomena/or exp Prenatal Exposure Delayed Effects/
7. 1 or 2 or 3 or 4 or 5 or 6
8. neonatal.mp. [mp = title, original title, abstract, name of substance word, subject heading word, unique identifier]
9. newborn*.mp. [mp = title, original title, abstract, name of substance word, subject heading word, unique identifier]
10. exp Infant, Newborn/ab, an, cl, co, di, et, gd, me, mi, mo, pa, pd, ph [Abnormalities, Analysis, Classification, Complications, Diagnosis, Etiology, Growth & Development, Metabolism, Microbiology, Mortality, Pathology, Pharmacology, Physiology]
11. infan*.mp. [mp = title, original title, abstract, name of substance word, subject heading word, unique identifier]
12. exp Infant/
13. fetus.mp. [mp = title, original title, abstract, name of substance word, subject heading word, unique identifier]
14. exp Fetus/
15. exp Fetal Development/
16. foetus.mp. [mp = title, original title, abstract, name of substance word, subject heading word, unique identifier]
17. exp Fetus/
18. foetal*.mp. [mp = title, original title, abstract, name of substance word, subject heading word, unique identifier]
19. 8 or 9 or 10 or 11 or 12 or 13 or 14 or 15 or 16 or 17 or 18
20. 7 or 19
21. diabet*.mp. [mp = title, original title, abstract, name of substance word, subject heading word, unique identifier]
22. exp Diabetes Mellitus, Type 2/or exp Diabetes Complications/or exp Diabetes Mellitus/
23. 21 or 22
24. hyperglyc?mi*.mp. [mp = title, original title, abstract, name of substance word, subject heading word, unique identifier]
25. exp Hyperglycemia/
26. glucose intoleran*.mp. [mp = title, original title, abstract, name of substance word, subject heading word, unique identifier]
27. exp Glucose Intolerance/or exp Glucose Tolerance Test/
28. obes*.mp. [mp = title, original title, abstract, name of substance word, subject heading word, unique identifier]
29. exp Obesity/
30. 24 or 25 or 26 or 27 or 28 or 29
31. 23 or 30
32. gestational diabet*.mp. [mp = title, original title, abstract, name of substance word, subject heading word, unique identifier]
33. exp Diabetes, Gestational/
34. 32 or 33
35. aborigin*.mp. [mp = title, original title, abstract, name of substance word, subject heading word, unique identifier]
36. exp Indians, North American/or exp Oceanic Ancestry Group/
37. indigen*.mp. [mp = title, original title, abstract, name of substance word, subject heading word, unique identifier]
38. exp Health Services, Indigenous/
39. 35 or 36 or 37 or 38
40. 20 and 31
41. 34 or 40
42. 39 and 41
43. 31 and 39
44. 20 and 39
45. native*.mp. or exp American Native Continental Ancestry Group/
46. (45 and 41) not 39

### Data collection

Citations identified by the search of the five databases will be downloaded into *Endnote*^© ^for removal of duplications, and all abstracts will be reviewed by two people to determine whether the publications potentially meet the criterion for inclusion in this review.

The full text of all publications that potentially meet the inclusion criterion will be reviewed by one assessor, and 10% will be independently reviewed by a second assessor for validation. When there is disagreement, a third person will make the final decision. The proportions of agreement between the first and second reviewer and between the first and final reviewer will be reported to provide an estimate of the degree of possible variation in interpretation.

Kappa scores will not be calculated, as there is a high rate of expected agreement that would require a larger sample size of co-reviewed papers than is feasible within the resource constraints of this review. All publications that do not meet the inclusion criterion, with reasons specified, will be available from the contact author on request.

### Data extraction

A spreadsheet will be prepared in *Microsoft Excel*^© ^to extract standardised data items from the included publications. One person will extract data from all the included publications, and 10% will be extracted independently by a second reviewer and checked for agreement. When there is disagreement, a third person will make the final decision. The proportions of agreement between the first and second reviewer and the first and final reviewer will be reported in order to provide an estimate of potential variation in interpretation. Again, kappa scores will not be calculated because of the high level of expected agreement.

Publications will be coded according to their focus on indigenous people in the following countries:

Australia (Aboriginal and/or Torres Strait Islander);

Canada (Cree, Saskatchewan Indian, Saskatoon Indian, Inuit, First Nation);

New Zealand (Maori);

United States (Navajo Indian, Papago, Athabaskan, Chamorro, Chippewa, Pima Indian, Tohono Indian, Zuni Indian, Hawaiin, Yup'ik Eskimo, Native Indian); and

"Other" (when more than one group or general reference is made).

Publications will be coded under one of the *research purposes *[28-30] and *study designs *[31] outlined in Table 2.

**Table 2:** Coding of study designs for a review of diabetes in pregnancy among indigenous women

Research purpose	Study design
**Descriptive**	*Descriptive *(cross-sectional survey or qualitative study) or *analytical *(cohort study, case-control study or cross-sectional study with control group)
**Intervention**	*Experimental *(control group) or *other*
**Measurement**	Screening test efficacy studies
**Other**	Reviews, opinions, guidelines, etc.

The following *outcome data *will be extracted: rationale, aims, number of people included, journal title, outcomes reported, main findings and conclusions, funding source, data source, GDM incidence, diagnostic criteria used and years of measurement.

The publication topics will be grouped according to the "*key criteria*" of the population-based screening framework [22] as: epidemiology and natural history of DIP, screening practice and rates, acceptability, efficacy and cost (measurement), adequate treatment pathways, systems for follow-up and "other" (including preventive interventions).

### Appraisal of external validity (generalisability)

The external validity will be appraised by coding the studies according to:

whether the population is rural, urban, remote or mixed; and

whether the data were collected from clinics, individual communities or broader populations.

### Appraisal of internal validity (risk for bias)

The *risk for bias *will be critically appraised with specific tools for different study designs. Intervention and measurement studies, reviews and qualitative descriptive studies will be appraised with the following tools:

*Intervention *studies will be appraised with the Centre for Evidence-Based Medicine (CEBM) Critical Appraisal Skills Programme (CASP) tool for intervention studies [32].

*Measurement *studies will be appraised with the CEBM CASP tool for studies of diagnostic accuracy [33].

*Reviews *will be appraised with the CEBM CASP tool for systematic reviews [34].

*Qualitative *research will be appraised with tools adapted from the Australian Department of General Practice and other experts for qualitative studies [35,36].

As no tool exists for appraising descriptive studies, we adapted an instrument from Strengthening the Reporting of Observational Studies in Epidemiology (STROBE) [37], a recent systematic review of descriptive studies [38] and a tool used by clinicians for rapid evidence appraisal [39]. Our tool was developed specifically for this review and pilot-tested with a sample of over 20 studies; it was subsequently modified, as some items could not be appraised (e.g. reporting bias) (see Table 3).

**Table 3:** Criteria for appraising risk for bias in descriptive studies

Type of risk	Low	Moderate	High
**Selection (sampling of population)**	Consecutive unselected population Sample from general population not a selected group Rationale for case and control selection explained Follow-up or assessment time appropriate	Sample selected from large population, but selection criteria not defined Sample selection ambiguous, but may be representative Eligibility criteria not described Rationale for case and control selection not described Follow-up assessment not appropriate Analysis to adjust for sampling strategy bias	Sample selection ambiguous, and sample unlikely to be representative Highly selected population, making it difficult to generalise findings
**Selection (sampling size) if non-significant results; otherwise NA**	Sample size calculation conducted and adequate	Sample size calculation not performed, but all eligible persons studied Sample size calculation performed, and reasons for not meeting sample size given	Sample size estimation unclear or only subsample studied
**Selection (participation rate)**	High participation rate (> 85%)	Moderate participation rate (70-85%)	Low participation rate (> 70%)
**Performance bias (outcome assessment)**	Diagnosis on basis of consistent criteria and direct examination	Assessment from hospital record or by questioning person Assessment from administrative database or register	Assessment from non-validated data or generic estimate from overall population
**Performance bias (confounding factors)**	Control for common confounders (e.g. risk factors)	Only certain confounders adjusted (e.g. age)	Not controlled for any confounders
**Performance bias (blinding) when outcome and exposure relations being assessed (e.g. effect of GDM on offspring)**	Outcome and exposure assessment independently blinded	Outcome and exposure assessment independently blinded	
**Performance bias (methods to control for bias)**	Analysis appropriate for type of sample (e.g. subgroup analysis/regression etc.)	Analysis did not account for common adjustment	Data confusing
**Attrition bias**	0-10% attrition All participants from initiation to final outcome assessment accounted for Sensitivity analysis conducted for missing data	11-20% attrition No sensitivity analysis for missing data	> 20% attrition and no sensitivity analysis for missing data
**Other: for comparative studies**	Comparison groups similar at baseline	Baseline characteristics of comparison groups not assessed	Baseline characteristics dissimilar

### Data analysis

Data will be analysed with *Microsoft Excel*^©^. The outcomes reported will include: the rates of publications per country and over time, the type of research that has been published, the internal validity and risks for bias identified in the research, the published findings relevant to population-based screening criteria and the generalisability of the findings.

A detailed "characteristics of included studies table" will be available on request, in which the included studies will be summarised in alphabetical order with: description of the population, study details, main findings and a summary of the risks for bias.

The conclusions about the strength of the evidence within each of the criteria will be clearly communicated to the reader by presenting a summary table adapted from the "GRADE" criteria, which is used to assess guideline evidence [27,40] (see Table 4).

**Table 4:** Classification of quality of evidence base for screening for gestational diabetes mellitus in indigenous women

GRADE symbol	Level of confidence in quality of evidence	Definition	Criteria
⊕ ⊕ ⊕ ⊕	**High**	We are confident the true effect lies close to the estimate of the effect.	One or more studies were appraised at low risk of bias.
⊕ ⊕ ⊕	**Moderate**	We are moderately confident in the effect estimate: the true effect is likely to be close to the estimate of the effect, but there is a possibility that it is substantially different.	No studies were appraised at low risk of bias, although one or more studies were appraised at moderate risk of bias.
⊕ ⊕	**Low**	Our confidence in the effect estimate is limited: the true effect may be substantially different from the estimate of the effect.	No studies were appraised as low or moderate risk of bias. One or more studies were appraised at high risk of bias.
⊕	**Very low**	We have very little confidence in the effect estimate.	The publications were not in a format that allowed appraisal of the effect estimate (e.g. opinion piece).

The summary of findings table will include columns for each of the screening criteria, an evidence statement for the main findings under each criterion (referenced), a strength of evidence symbol and a comment about the generalisability of each statement (i.e. remote, rural, urban or mixed communities and country).

## Discussion

This paper outlines a process for systematic review and appraisal of both the internal and external validity of the published literature and for presenting it in a framework to inform decisions about population-based screening for GDM among indigenous women in Australia, Canada, New Zealand and the United States.

The heterogeneity of the study designs and the heterogeneity between studies of the same design (e.g. different diagnostic criteria for GDM and different criteria for identification of indigenous status both between countries and between studies in descriptive prevalence studies) will preclude a meta-analysis of the studies. Nevertheless, the authors consider it valuable to present a narrative synthesis of the relevant research that has been published under each of the population-based screening criteria.

Calculating kappa scores was not feasible, as the high rate of expected agreement for some criteria would have required co-review by a second person of approximately half of the 123 included publications. Resources permitting, it would be desirable to have all the data extracted by two people.

The inclusion criterion for this review does not include potentially relevant evidence on other population groups or topics such as cancer screening. We do not propose that the evidence from the published academic literature on a specific topic for population groups with some similarities would, in itself, provide sufficient information to enable informed decision-making. However, this review should be a useful resource to contribute to the evidence base for developing interventions in this area.

The vast majority of the studies are observational, descriptive and quantitative, for which there are no commonly used or widely accepted critical appraisal tools. The STROBE guidelines provide a useful reference point; however, they are designed for *reporting *rather than for the *methods *of research. The tool presented here for appraising quantitative descriptive studies was adapted for the studies in this review. Specific consideration was paid to the potential for selection bias, which can be from a non-representative source, and the criteria used for identifying indigenous status (e.g. blood quantum specifications). As we were unable to develop clear objective criteria for assessing analytical bias, we adapted a tool that was already available [39]. As there was insufficient information to assess reporting bias, this aspect was removed after pilot-testing

One of the common criticisms of academic literature is that the criteria for internal validity are too restrictive and relevant evidence is not presented in a way that is useful for policy-makers [26]. A strength of this review is that a relevant framework is described, with criteria [22] that define the range of study designs that should be included. While consideration of a broad range of study designs presents methodological challenges for systematic reviews of the literature, the method described here presents a means for rigorous appraisal of the internal and external validity of the included studies.

The method for our review was adapted to allow inclusion of the perspectives and experiences of indigenous women and communities, which are clearly relevant to policy-makers. At the time of writing, we were unable to find any structured research about DIP that was explicitly reported from an indigenous perspective, thus highlighting a critical gap, or "perspective bias", in the research. This gap may be due to a number of factors, such as an under-representation of indigenous people in academic institutions, lack of trust between indigenous communities and academic institutions and the notion that "objectivity" is desirable in public health research, thus negating the validity of perspectives.

Indigenous women in Australia, Canada, New Zealand and the United States have one of the highest prevalence rates of T2DM in the world; therefore, there is consensus that they should be offered changes to current screening practice in the near future. However, there are unique social and health considerations for this vulnerable population group which requires active collaboration and careful reflection on the existing evidence base. Policy-makers need information presented in a format that is useable and that includes *all *the relevant data for which both the internal and external validity have been rigorously appraised and the evidence gaps clearly identified, to ensure that decisions made on the basis of limited evidence are carefully evaluated.

This paper has described a process of adapting rigorous systematic review methods to include different study designs, in order to produce relevant evidence for a contemporary discussion about screening policies for DIP among pregnant indigenous women.

## Abbreviations

DIP: diabetes in pregnancy; GDM: gestational diabetes mellitus; T2DM: type 2 diabetes mellitus.

## Competing interests

The authors declare that they have no competing interests.

## Authors' contributions

CC prepared the protocol, developed and piloted the appraisal tools in collaboration with local experts, and drafted the manuscript. DY developed and pilot-tested the appraisal tools, reviewed 10% of the full texts to confirm inclusion and exclusion of studies and to check data extraction quality, and provided feedback on drafts. HL developed and pilot-tested the appraisal tools, acted as the third reviewer when there was a discrepancy in appraisals, and provided feedback on drafts. EW co-reviewed abstracts of all search results for included studies and provided feedback on drafts. BM assisted in development of appraisal tool and provided feedback on drafts. BO had input into the review protocol and provided feedback on drafts. JO had input into the review protocol and provided feedback on drafts. SE had input into review protocol, advised on relevant indigenous comparisons, and provided feedback on drafts. All authors read and approved the final manuscript.

## Authors' information

This paper was written by Catherine Chamberlain as part of a PhD program investigating gestational diabetes among Aboriginal and Torres Strait Islander women in Australia.

## Pre-publication history

The pre-publication history for this paper can be accessed here:

http://www.biomedcentral.com/1471-2393/11/104/prepub
